# BMP signaling in cancer stemness and differentiation

**DOI:** 10.1186/s13619-023-00181-8

**Published:** 2023-12-05

**Authors:** Wei Zhou, Kun Yan, Qiaoran Xi

**Affiliations:** 1 State Key Laboratory of Molecular Oncology, MOE Key Laboratory of Protein Sciences, School of Life Sciences, Tsinghua University, Beijing, 100084 China; 2 Tsinghua-Peking Center for Life Sciences, School of Life Sciences, Tsinghua University, Beijing, 100084 China; 3 Joint Graduate Program of Peking-Tsinghua-NIBS, Tsinghua University, Beijing, China

**Keywords:** BMP signaling, Cancer stem cell, Stemness, Differentiation

## Abstract

The BMP (Bone morphogenetic protein) signaling pathway plays a central role in metazoan biology, intricately shaping embryonic development, maintaining tissue homeostasis, and influencing disease progression. In the context of cancer, BMP signaling exhibits context-dependent dynamics, spanning from tumor suppression to promotion. Cancer stem cells (CSCs), a modest subset of neoplastic cells with stem-like attributes, exert substantial influence by steering tumor growth, orchestrating therapy resistance, and contributing to relapse. A comprehensive grasp of the intricate interplay between CSCs and their microenvironment is pivotal for effective therapeutic strategies. Among the web of signaling pathways orchestrating cellular dynamics within CSCs, BMP signaling emerges as a vital conductor, overseeing CSC self-renewal, differentiation dynamics, and the intricate symphony within the tumor microenvironment. Moreover, BMP signaling’s influence in cancer extends beyond CSCs, intricately regulating cellular migration, invasion, and metastasis. This multifaceted role underscores the imperative of comprehending BMP signaling’s contributions to cancer, serving as the foundation for crafting precise therapies to navigate multifaceted challenges posed not only by CSCs but also by various dimensions of cancer progression. This article succinctly encapsulates the diverse roles of the BMP signaling pathway across different cancers, spanning glioblastoma multiforme (GBM), diffuse intrinsic pontine glioma (DIPG), colorectal cancer, acute myeloid leukemia (AML), lung cancer, prostate cancer, and osteosarcoma. It underscores the necessity of unraveling underlying mechanisms and molecular interactions. By delving into the intricate tapestry of BMP signaling’s engagement in cancers, researchers pave the way for meticulously tailored therapies, adroitly leveraging its dualistic aspects—whether as a suppressor or promoter—to effectively counter the relentless march of tumor progression.

## Background

The BMP (Bone morphogenetic protein) signaling pathway plays a crucial role in various aspects of metazoan biology. From embryonic development to tissue homeostasis and disease progression, the BMP signaling exerts a profound influence on cellular processes and organismal physiology (Massagué 2012). The outcome of BMP signaling response in cancer is highly context-dependent. The regulatory cytokine BMP exerts tumor-suppressive effects that cancer cells must evade to undergo malignant evolution (Cai et al. 2012; Guo and Wang 2009; Owens et al. 2015). Paradoxically, BMP also modulates processes such as cell invasion, stemness, and modification of the microenvironment that cancer cells may exploit to their advantage (Martínez et al. 2017; Wang et al. 2019; Yan et al. 2012).

Cancer stem cells (CSCs), also known as tumor-initiating cells, are a small subpopulation of quiescent, pluripotent, self-renewing neoplastic cells that were first identified in hematologic tumors and later in solid malignancies (Bao et al. 2006; Chen et al. 2012; Shibue and Weinberg 2017). CSCs possess stem-like properties and contribute to tumor initiation, progression, and resistance to therapy. Their role in tumor resistance to chemotherapy and radiation treatment, as well as recurrence, has garnered significant research interest. CSCs are thought to be preserved as a small population through self-renewal, and to generate more differentiated progenies that constitute the bulk of the tumor mass (Kreso and Dick 2014). In addition to providing the driving force for tumor growth and maintenance, CSCs have been shown to be more resistant to existing anticancer therapies, consistent with their role in relapse after therapy. Accordingly, transcriptional signatures of CSCs are predictive of overall patient outcome, supporting their clinical relevance.

The expanding array of aberrant signaling pathways, including BMP, Hippo, Hedgehog, JAK/STAT, Wnt, Notch, PI3K/PTEN, and NF-κB, distinctly regulates the sustenance of cancer stem cells (CSCs) (Clara et al. 2020; Takebe et al. 2015; Yang et al. 2020). While governing normal stem cell equilibrium, these pathways often experience anomalous activation or repression in CSC contexts. The BMP antagonist COCO plays a pivotal role in modulating the reawakening of dormant metastatic breast cancers linked to CSCs in the lung, whereas BMP signaling itself exerts suppressive effects (Gao et al. 2012). YAP/TAZ activation equally emerges as significant, instigating CSC attributes, fueling proliferation, encouraging chemoresistance, and driving metastasis (Zanconato et al. 2016). The JAK/STAT pathway, a pivotal player, drives CSC-mediated metastasis and proliferation in various cancers, including colon cancer (Calon et al. 2012), glioblastoma (Sherry et al. 2009), and breast cancer (Zhou et al. 2007).

Importantly, these pathways form a complex interwoven network of signaling mediators, intricately interacting and fostering a labyrinthine cross-talk. This interconnected web underscores the significance of understanding not only each pathway’s distinct role but also their collaborative dynamics. Together, they intricately shape the landscape of CSC regulation and cancer progression.

Understanding the biology of CSCs and their interactions with the tumor microenvironment is of paramount importance in the pursuit of effective therapies for intractable tumors. The intricate functioning of the BMP signaling has been demonstrated to play a crucial role in regulating CSC self-renewal, differentiation, and the modulation of the tumor microenvironment in various cancer types (Table 1). Moreover, the influence of BMP signaling extends beyond CSCs, intricately regulating cellular migration, invasion, and metastasis across different tumors.

**Table 1:** The BMP family members and their functions in stemness and differentiation of various cancers

BMP members	Cancer type	Phenotype	References
BMP2	Ovarian	Promote growth and stemness	Choi et al. 2015
	Lung	Enhance stemness	Husanie et al. 2022
	Breast	Induce EMT and stemness	Huang et al. 2017; Zhang et al. 2018
BMP4	Lung	Promote differentiation	Lee et al. 2014b
	DIPG	Inhibit stemness and growth	Sun et al. 2022
	GBM	Promote differentiation	Savary et al. 2013
BMP5	Colorectal	Inhibit stem cell-like properties, proliferation, migration, invasion	Chen et al. 2018
	Breast	Reduce stemness	Jin et al. 2022
BMP6	Prostate	Induce differentiation	Lee et al. 2011
	Medulloblastoma	Induce differentiation	Armandari et al. 2021
BMP7	Colorectal	Antiangiogenic and prodifferentiation	Veschi et al. 2020
	Glioma	Promote differentiation and reduce stemness	Caja et al. 2018; Tate et al. 2012; Tso et al. 2015
BMP8	Gastric	Reduce differentiation	Wisnieski et al. 2017
BMP9	GBM	Induce differentiation	Porcù et al. 2018
GDF6	Melanoma	Prevent differentiation and cell death, promote tumor growth	Gramann et al. 2019; Venkatesan et al. 2018
AMH	Endometrial	Activate differentiation and inhibit tumor growth	Fortner et al. 2017
BMPR1A	CRC	Inhibit stem cell activation	Kodach et al. 2011
BMPR1B	GSC	Astroglial differentiation in GSCs	Lee et al. 2008
ACVR1A	DIPG	Promote stemness and progenitor cell arrest	Fortin et al. 2020; Hoeman et al. 2019
BMPR2	NSCLC	Increase cell migration and invasiveness	Wu et al. 2022

The complex nature of BMP signaling in cancer underscores the need to comprehend its effects within the cellular context and the tumor microenvironment. Given the interplay between the tumor-suppressive and tumor-promoting aspects of BMP signaling, it is imperative to grasp the underlying mechanisms and specific molecular interactions involved. Thus, the objective of this article is to provide a concise overview that highlights the diverse roles of the BMP signaling in various types of cancers.

## Basics of BMP signaling pathway

The core BMP signaling components are largely conserved across metazoans (Massagué 2012). The BMP signaling pathway comprises an extensive repertoire of ligands, with more than 20 identified members. These ligands can be classified based on their nucleotide or amino acid similarities. Among the noteworthy ligands within the pathway are BMPs 2, 4, 6, 7, 9, and 15, along with growth differentiation factors (GDFs) 5 and 9, and anti-Müllerian hormone (David and Massagué 2018) (Table 2). Initially derived from demineralized bone matrix, BMPs exhibit remarkable capacity to induce bone formation (Yang et al. 2020). These ligands belong to the transforming growth factor (TGF)-β superfamily (Derynck et al. 2021; Liu et al. 2021).

**Table 2:** The BMP family members and their receptors

Ligand	Type I receptor	Type II receptor	Co-receptor
BMP2	BMPR1A, BMPR1B	ACVR2, ACVR2B, BMPR2	RGM
BMP4	BMPR1A, BMPR1B	ACVR2, ACVR2B, BMPR2	
BMP5	ACVR1A, BMPR1A, BMPR1B	ACVR2, ACVR2B, BMPR2	
BMP6	ACVR1A, BMPR1A, BMPR1B	ACVR2, ACVR2B, BMPR2	RGM
BMP7	ACVR1A, BMPR1A, BMPR1B	ACVR2, ACVR2B, BMPR2	
BMP8	ACVR1A, BMPR1A, BMPR1B	ACVR2, ACVR2B, BMPR2	
BMP9	ALK1	ACVR2, BMPR2	Endoglin
BMP10	ALK1	ACVR2, BMPR2	Endoglin
BMP15	BMPR1B	BMPR2	
BMP3^a^	-	-	
BMP3B	-	-	
GDF5	BMPR1A, BMPR1B	ACVR2, ACVR2B, BMPR2	
GDF6	BMPR1A, BMPR1B	ACVR2, ACVR2B, BMPR2	
GDF7	BMPR1A, BMPR1B	ACVR2, ACVR2B, BMPR2	
AMH	ACVR1A, BMPR1A	AMHR2	
GDF15	GFRAL^b^		

*BMP *Bone morphogenetic protein, *BMPR *Bone morphogenic protein receptor, *ACVR *Activin receptor, *RGM *Repulsion guidance molecules, *GDF *Growth and differentiation factor, *AMH *Anti-Muellerian hormone, *AMHR *Anti-Muellerian hormone receptor; -, not applicable. ^a^BMP3 antagonizes other BMPs. ^b^GDF15 is a distant member of the TGF-β and BMP family. It signals through a receptor called glial-derived neurotrophic factor receptor alpha-like (GFRAL)

During embryogenesis, BMP signaling participates in key developmental events such as dorsal-ventral patterning, mesoderm and ectoderm specification, as well as organogenesis (Jia et al. 2012). It regulates cell fate determination, proliferation, and differentiation, guiding the formation of diverse tissues and organs throughout the body (Bier and De Robertis 2015; Salazar et al. 2016; Wu et al. 2016). Furthermore, BMP signaling is involved in maintaining tissue homeostasis in adult organisms by influencing cell growth, survival, and regeneration in various organs and tissues, including bone, muscle, skin, and the central nervous system (Agius et al. 2010; Bier and De Robertis 2015; Liu and Niswander 2005; Stevens et al. 2017; Zinski et al. 2018).

Beyond development and tissue maintenance, the BMP signaling has emerged as a crucial player in disease contexts. Dysregulation of BMP signaling has been implicated in several pathological conditions, including cancer, cardiovascular diseases, and developmental disorders (Davis et al. 2016; Martínez et al. 2017; Morrell et al. 2016; Palencia-Desai et al. 2015; Walton et al. 2016; Wang et al. 2019; Yan et al. 2012). Aberrant activation or inhibition of BMP signaling can lead to uncontrolled cell proliferation, abnormal tissue remodeling, and functional impairments in affected tissues (Martínez et al. 2017; Wang et al. 2019; Yan et al. 2012).

**Fig. 1:**
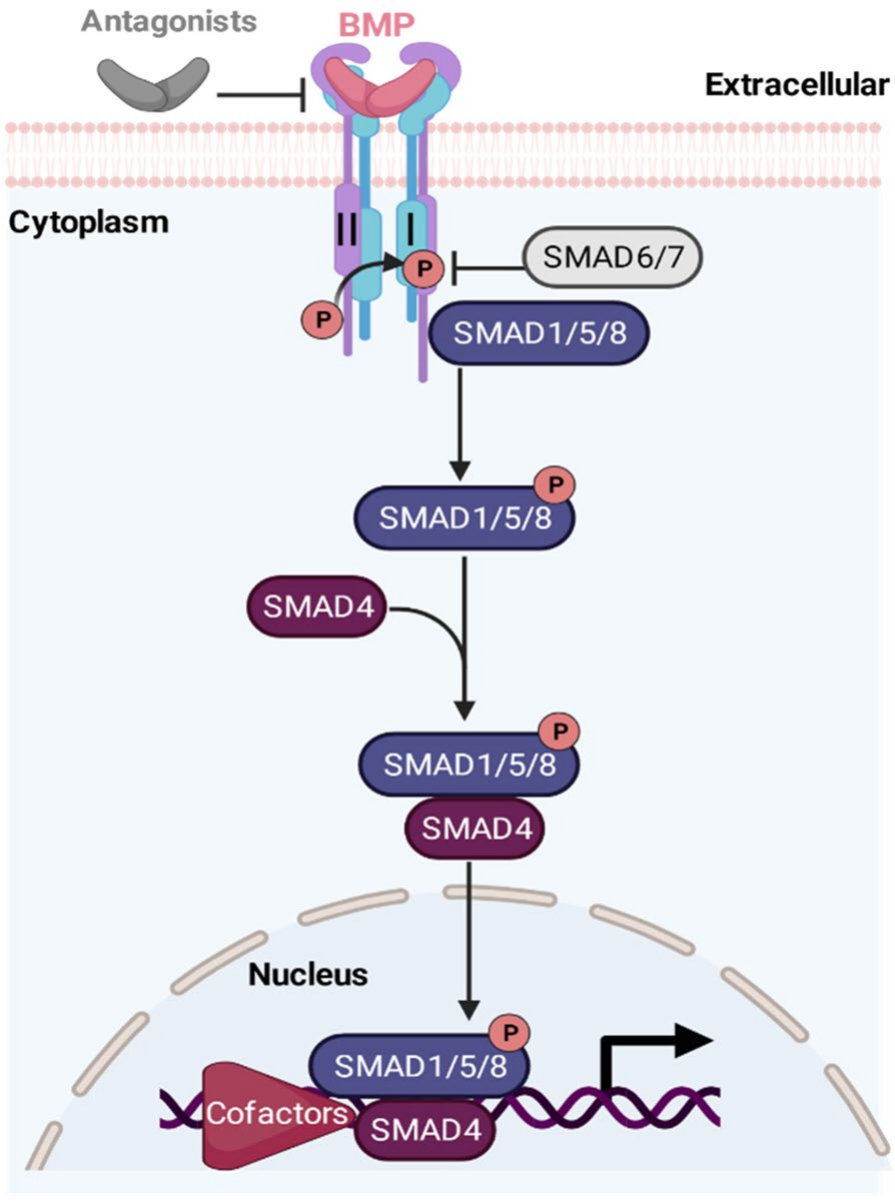
Schematic illustration of BMP signaling pathway. Activation of this pathway occurs when BMP ligand dimers bind to two homologous type II receptors, facilitating the formation of a tetramer with the two type I receptors. The type II receptor kinase, constitutively active, phosphorylates specific serine residues in the type I receptors, leading to their activation. There are four type I BMP receptors: ALK1 (ACVRL1), ALK2 (ACVR1), ALK3 (BMPRIA), and ALK6 (BMPRIB), and three type II BMP receptors: BMP receptor type II (BMPR2), activin type II receptor A (ACVR2A), and activin type II receptor B (ACVR2B). The subsequent steps involve the activation of type I receptors, leading to the phosphorylation of receptor-regulated SMADs (R-SMADs) transcription factors, specifically SMAD1/5/8. In contrast, TGFβ signaling involves SMAD2/3. The R-SMADs form a heteromeric complex with the co-SMAD, SMAD4, and translocate into the nucleus to regulate target gene expression transcriptionally. Created with BioRender.com

The multifaceted nature of BMP signaling is attributed to its intricate network of ligands, receptors, and downstream effectors. BMP ligands bind to specific transmembrane receptors, initiating a cascade of intracellular events that lead to the activation of downstream effectors, including SMAD proteins (Agnew et al. 2021; Gaarenstroom and Hill 2014; Gomez-Puerto et al. 2019). Once activated, these effectors translocate to the nucleus and modulate gene expression, thereby orchestrating the cellular responses associated with BMP signaling (David and Massagué 2018; Massagué et al. 2005) (Fig. 1).

Regulation of BMP signaling involves multiple levels of control, encompassing intricate mechanisms that govern the activation, expression, and degradation of key components within the signaling pathway. At the transcriptional level, the expression of BMP ligands, receptors, and downstream effectors is tightly regulated by a variety of transcription factors and co-regulators (Huse et al. 1999; Massagué et al. 2005). Post-translational modifications, such as phosphorylation and ubiquitination, dynamically modulate the activity and stability of BMP receptors, thereby influencing the strength and duration of BMP signaling (Massagué et al. 2005; Shen et al. 2014). Additionally, extracellular regulators, including antagonists and binding proteins, act as molecular rheostats, fine-tuning the availability and localization of BMP ligands (Walsh et al. 2010). Crosstalk with other signaling pathways further adds another layer of complexity, allowing for intricate regulatory networks that shape the precise outcomes of BMP signaling in diverse biological contexts. Overall, the regulation of BMP signaling involves a sophisticated interplay of multiple levels of control, ensuring precise and context-dependent responses to this essential cellular pathway.

## BMP signaling in Glioblastoma Multiforme (GBM)

Glioblastoma multiforme (GBM) is a malignant brain tumor in adults, is challenging to treat due to its diverse cellular populations with varying transcriptional profiles, morphology, invasive potential, tumorigenicity, and drug sensitivity (Aldape et al. 2015; Jackson et al. 2019; Khan et al. 2023; Krishna et al. 2023; Miska and Chandel 2023; Vescovi et al. 2006). Glioblastoma stem cells (GSCs), functionally defined by their self-renewal and tumor-propagating ability, exhibit high resistance to radiation and chemotherapy, resulting in poor patient survival (Ranjan et al. 2023; Singh et al. 2004). Neural stem cells (NSCs) share regulatory mechanisms of self-renewal capacity and long-term proliferative potential with GSCs, but undergo terminal differentiation to generate different lineages of mature cells, including astrocytes, oligodendrocytes, and neurons, for tissue homeostasis (Blanpain and Fuchs 2014).

Research findings have demonstrated the indispensable role of TGF-β signaling in upholding the stem cell-like attributes and tumorigenic prowess of GSCs (Ikushima et al. 2009). Perturbation of TGF-β signaling leads to the attenuation of GSCs’ tumorigenic potential, while concurrently triggering the activation of SOX2 and SOX4 through TGF-β signaling, thus perpetuating GSCs’ stemness (Ikushima et al. 2009). Conversely, BMP signaling assumes the role of a tumor suppressor within the context of GBM. Stimulation of BMP signaling coerces GSCs towards adopting an astroglial differentiation fate, consequently impeding the progression of tumor growth (Lee et al. 2008; Piccirillo et al. 2006).

BMP signaling exerts its tumor suppressive function in GBM through the upregulation of *SNAI1* (also called *SNAIL*) and *DLX2* (Raja et al. 2017; Savary et al. 2013). *SNAI1* induction is correlated with GFAP upregulation and GSC differentiation, indicating SNAI1 is indispensable for BMP signaling-induced differentiation. However, *SNAI1* overexpression only partially phenocopies the BMP signaling response in GSC cells, as BMP signaling downregulates Nestin expression, which SNAI1 does not (Savary et al. 2013). *DLX2* is highly induced upon BMP signaling, and overexpression of *DLX2* significantly decreases GSC cell viability and induces apoptosis. Knockdown of DLX2 blocks the inhibitory effects of BMP signaling on GSCs. Clinically, patients with high expression of *DLX2* (BMP signaling targets) survive longer than patients with low expression of *DLX2* (Raja et al. 2017).

Despite the tumor suppressive role traditionally associated with BMP signaling in GSCs, human gliomas contain high levels of BMP ligands. A study by Yan et al. revealed that BMP signaling is more active in non-GSCs compared to GSCs. Interestingly, GSCs secrete elevated levels of the BMP antagonist Gremlin1, promoting their stemness by blocking BMP signaling. Moreover, overexpressing Gremlin1 in non-GSCs enhances their tumor-initiating capacity, and it stimulates cell cycle progression in GSCs by inhibiting p21 activity. These findings highlight the complex interplay between BMP signaling, Gremlin1, and distinct cell populations in gliomas (Yan et al. 2014).

In summary, BMP signaling has shown a tumor suppressor role in GBM, particularly in GSCs, making it a potential target for therapeutic intervention. Previous studies have explored methods such as local delivery of BMP4-saturated beads or intracranial administration of BMP4-expressing viruses, which have demonstrated improved survival in preclinical models. However, it is important to carefully balance BMP signaling activation to avoid immune misregulation and potential tumor progression in advanced cancers. Therefore, future research should focus on precise analysis of this signaling pathway in GBM and identify specific downstream targets for inclusion in clinical trials. Overall, augmenting BMP signaling in GSCs holds promise as a therapeutic strategy for GBM treatment.

## BMP signaling in diffuse intrinsic pontine glioma (DIPG)

Diffuse intrinsic pontine glioma (DIPG) is a devastating brainstem tumor located in the pons, accounting for 10–15% of all pediatric brain tumors, with a median survival of only 9–12 months (Jones et al. 2017; Wong et al. 1999). Recent large-scale genomic and epigenomic sequencing studies have shed light on driver mutations and their associated genomic and epigenomic landscape in DIPG patients. Nearly 80% DIPG patients carry a characteristic mutation of lysine 27 to methionine (K27M) in histone H3.3 and H3.1 (Bocciardi et al. 2009; Buczkowicz et al. 2014; Fontebasso et al. 2014; Khuong-Quang et al. 2012; Taylor et al. 2014a, b; Wu et al. 2012, 2014).

Approximately 20% of DIPG cases harbor recurrent ACVR1 mutations co-occurred with H3.1K27M, which encode the BMP type I receptor, also known as ALK2 (Bocciardi et al. 2009; Buczkowicz et al. 2014; Fontebasso et al. 2014; Taylor et al. 2014a, b; Wu et al. 2014). These mutations, located in the GS domain (R206H) and the protein kinase domain (R258G, G328V, G328E, and G356D), cause ligand-independent constitutive activation of the BMP signaling pathway, leading to the phosphorylation of SMAD1/5/8 (Atsuta and Takahashi 2016; Fontebasso et al. 2014; Hegarty et al. 2013; Shen et al. 2009; Shore et al. 2006; Stevens et al. 2017). Notably, these mutations also occur in the congenital malformation syndrome fibrodysplasia ossificans progressiva (FOP), where they activate the BMP signaling pathway, resulting in the transformation of soft tissues into bone (Kaplan et al. 2020; Shore et al. 2006; Taylor et al. 2014a, b).

Studies have shown that three of the most common ACVR1 mutants (R206H, G328V, and G328E) alone are not sufficient to induce DIPGs (Fortin et al. 2020; Hoeman et al. 2019). The combination of ACVR1 mutations with H3.1K27M and p53 deletion causes glioma-like lesions with a mesenchymal phenotype, though not enough to induce gliomagenesis (Fortin et al. 2020). Full gliomagenesis requires activation of PDGFRA signaling (Hoeman et al. 2019). Moreover, expression of *Acvr1*^*G328V*^ in murine oligodendroglial cells causes neurological anomalies. *Acvr1*^*G328V*^ induces ligand-independent BMP signaling activation and upregulates PDGFRA to block oligodendrocyte differentiation. Thus, *Acvr1*^*G328V*^ cooperates with *Hist1h3b*^*K27M*^ and *Pik3ca*^*H1047R*^ to induce high-grade diffuse gliomas (Fortin et al. 2020). These results suggest that ACVR1 mutations, which cause BMP signaling activation, drive tumorigenesis of DIPG and arrest this glioma at progenitor cell states.

The elevated BMP signaling activity implicated in tumorigenesis of ACVR1 mutant and H3.1K27M subtype DIPG suggests that targeting ACVR1 may hold promise as a therapeutic strategy. ACVR1-targeting drugs, including LDN-214,117, LDN-193,189, and LDN212854, have shown potential in preclinical studies for treating this specific subtype of DIPG (Carvalho et al. 2019; Hoeman et al. 2019). They selectively inhibit DIPG cell growth, reduce phospho-SMAD1/5/8 levels, block *ID1* expression, and demonstrate anti-tumor efficacy both in vitro and in vivo (Carvalho et al. 2019). E6201, a previously defined covalent inhibitor of MEK1/2, has been identified to associate with ACVR1, and inhibits BMP ligand-stimulated phosphorylation of SMAD1 (Fortin et al. 2020). E6201 demonstrated anti-tumor efficacy in DIPG cells and *Acvr1*^*G328V*^ DIPG mouse models. In summary, drugs targeting the BMP signaling pathway, especially ACVR1, may provide clinical options for DIPG patients with ACVR1 mutations.

Thus far, the ACVR1 mutation subtype of DIPG has received a significant amount of research attention, despite the fact that only 20% of DIPG patients carry ACVR1 mutations. Analysis of the active enhancer landscapes in H3.1K27M and H3.3K27M DIPG indicates that the differentially accessible enhancer elements of H3.3K27M DIPG are enriched in negative regulation of the BMP signaling compared with H3.1K27M DIPG (Nagaraja et al. 2019). Recent investigations have highlighted the diminished activity of BMP signaling in H3.3K27M ACVR1 WT subtype DIPG. Notably, BMP4 ligands have been found to exert robust tumor-suppressive effects on this particular subtype of DIPG. These effects are achieved by facilitating the transition of DIPG tumor cells from a prolonged stem-cell-like state to a state of differentiation, primarily through epigenetic regulation of CXXC5 (Sun et al. 2022). Moreover, the tumor suppressive effects of BMP signaling on ACVR1 wild-type (WT) and H3.3K27M subtype DIPG are supported by clinical evidence showing that patients with high expression of *CXXC5* or *ACVR1* tend to have a better prognosis, while low expression of *CHRDL1* is associated with improved outcomes (Sun et al. 2022).

Thus, these findings unveil four potential therapeutic opportunities for H3.3K27M ACVR1 WT subtype DIPG by enhancing BMP signaling: (1) targeting CHRDL1, an antagonist of the BMP pathway, could be achieved by inhibiting its activity, potentially utilizing a neutralizing antibody against CHRDL1; (2) inhibiting FPKBP12, a negative regulator of BMP receptors, through degradation or blocking strategies such as PRO-TAC technology or FK50663, could activate BMP signaling and impede tumor growth; (3) augmenting CXXC5 activity, a positive regulator of BMP signaling, could be pursued to suppress tumor growth; (4) HDACis drugs, which have exhibited anti-tumor efficacy in DIPG and can positively regulate BMP signaling, holds promise for improved therapeutic outcomes. These approaches provide encouraging avenues for the development of novel therapies targeting this aggressive cancer.

In sum, given the contrasting roles of BMP signaling in the two subtypes of DIPG, it is essential to explore distinct therapeutic strategies tailored to each subtype.

## BMP signaling in colorectal cancer

The intestinal mucosa harbors self-renewing stem cells in the crypt base and differentiated cells in the villus, which are tightly regulated by gradients of BMP and WNT signaling pathways (Beumer et al. 2022; Kraiczy et al. 2023; McCarthy et al. 2020). Stem cell maintenance and division are facilitated by high levels of WNT signaling and low levels of BMP signaling in the crypt base, while differentiation and apoptosis of daughter cells in the top villus are driven by low levels of WNT signaling and high levels of BMP signaling (Qi et al. 2017; van den Brink and Offerhaus 2007). The coordination between WNT and BMP signaling is necessary and sufficient to maintain intestinal stem cells self-renewal (Barker et al. 2007; Li et al. 2018; Wang and Chen 2018), while abnormal activation of WNT signaling and loss of BMP signaling would contribute to the development of colorectal carcinogenesis (Zhang and Que 2020).

The BMP signaling pathway is imperative in maintaining intestinal epithelial homeostasis and preventing the development of colorectal cancer (CRC). BMP signaling promotes intestinal differentiation while inhibiting stem cell activation. However, germline mutations in BMPR1A and SMAD4 are responsible for familial juvenile polyposis syndrome, which carries a high lifetime risk of CRC (Kodach et al. 2011). Genome-wide association studies have identified mutations in other members of the BMP signaling pathway that are associated with an increased risk of CRC, including BMP2, BMP4, GREM1, and SMAD7 (Broderick et al. 2007; Houlston et al. 2008), which can disrupt normal BMP signaling in the intestinal mucosa. The loss of BMP signaling leads to the formation of ectopic crypts, juvenile polyps, and eventually tumors (Haramis 2004).

Studies on transgenic mice have revealed that BMP signaling inhibits crypt fossa formation and polyp growth by suppressing WNT signaling (Haramis 2004; He et al. 2004) and controls crypt division by inhibiting stem cell self-renewal and replication (Haramis 2004). BMP signaling is typically intact in normal colonic epithelial cells and various types of adenomas but frequently inactivated in cancer cells (Kodach et al. 2008a, b). BMP4 treatment can increase PTEN levels, inhibit the PI3K/AKT pathway, antagonize the proliferative effects of WNT, and induce the differentiation of colorectal cancer stem cells (Lombardo et al. 2011). Thus, BMP signaling is considered a vital suppressor of intestinal tumorigenesis.

The secretion of BMP antagonists, such as Gremlin1, Gremlin2, and Noggin, is also tightly regulated in the intestine (Stzepourginski et al. 2017). These antagonists, which are derived exclusively from subcrypt myofibroblasts, act locally within the basal stem cell of the crypt to inhibit BMP signaling and maintain stemness (Kosinski et al. 2007). A duplication of approximately 40 kb upstream of the GREM1 gene leads to hereditary mixed polyposis syndrome (HMPS), an autosomal dominant disorder that predisposes untreated patients to develop colorectal cancer at a median age of 47 years (Jaeger et al. 2012). Aberrant epithelial expression of GREM1 disrupts the intestinal morphogenetic gradient and alters daughter cell fate, initiating colonic tumorigenesis from cells outside of the crypt base stem cell niche (Davis et al. 2015). Inhibition of BMP signaling in epithelial cells by transgenic overexpression of Noggin leads to the formation of ectopic crypts and polyps in the mouse intestine, mimicking the intestinal histopathology of juvenile polyposis (Batts et al. 2006; Haramis 2004).

Transcription factors also play a pivotal role in BMP signaling regulation. BMP signaling exerts a growth-suppressive effect in HT-29 through upregulation of RUNX3, which binds with T-cell factor 4 (TCF4) to form a complex with β-catenin. This complex negatively regulates WNT signaling by inhibiting the transcriptional activity of β-catenin/TCF4 on promoters of WNT target genes like the oncogene *c-MYC*. However, TGF-β has no effect on RUNX3 expression (Lee et al. 2010). Interestingly, elevated expression of BMP4 is specific to colorectal cancer, while other BMPs are not elevated in colorectal cancer cells (Yokoyama et al. 2017). Additionally, studies have found that BMP2 is silenced by promoter hypermethylation in a subgroup of CRCs. Statin treatment can inhibit DNA methyltransferase activity, demethylate the promoters of BMP2, and promote a shift from a stem-like state to a more differentiated state in CRCs (Kodach et al. 2011).

The potential of BMP signaling in the treatment of CRC has been explored, and it has been found that BMP signaling enhances the cytotoxic effects of chemotherapy, suggesting that combining BMP4 administration with current standard chemotherapy could provide clinical benefits for CRC patients (Lombardo et al. 2011). Furthermore, BMP2 has been identified as a differentiating and radiosentizing agent for colorectal cancer stem cells, suggesting that restoring the BMP signaling pathway may offer novel therapeutic approaches for colorectal cancer (Mahmoudi et al. 2023). In light of the systemic effects of BMP signaling on patients, future clinical strategies should focus on targeting specific members of BMP pathway to maximize benefits.

## BMP signaling in acute Myeloid Leukemia (AML)

Acute myeloid leukemia (AML) is a highly aggressive hematological malignancy that is characterized by the uncontrolled proliferation of hematopoietic stem/progenitor cell (HSPCs) and blockage of myeloid differentiation (De Kouchkovsky and Abdul-Hay 2016; Döhner et al. 2015). The self-renewing leukemia stem cells (LSCs), which share properties with normal hematopoietic stem cells (HSCs) producing normal blood cells, initiate and sustain AML cells (Gal et al. 2006). The WNT and BMP signaling pathways have been implicated in the aberrant proliferation of AML cells during disease progression (Gruber et al. 2012; Raymond et al. 2014; Voeltzel et al. 2018). Studies have revealed that activation of BMP signaling maintains progenitors in an undifferentiated state, resulting in therapeutic resistance (Gruber et al. 2012; Raymond et al. 2014; Voeltzel et al. 2018), while others have reported that BMP signaling inhibits growth and induces differentiation of myeloid progenitors and AML cells (Imai et al. 2001; Wang et al. 2006).

Increased BMP signaling has been shown to induce differentiation of CD34^+^ cells into megakaryocytes (Jeanpierre et al. 2008). In contrast, fusion-positive acute megakaryoblastic leukemias (AMKLs) with the CBFA2T3-GLIS2 fusion exhibit altered expression of BMP, SHH, and WNT pathway genes, particularly BMP2 and BMP4 (Gruber et al. 2012). BMP2/4 act in an autocrine or paracrine manner to promote growth and induce a megakaryocytic lineage phenotype in AMKL blasts and hematopoietic progenitors (Crispino and Le Beau 2012; Gruber et al. 2012). Another study has identified intrinsic and extrinsic upregulation of the BMP signaling in AML patients at diagnosis. They found BMP4 controls the expression of the survival factor ΔNp73 through its binding to BMPR1A, which results in the direct induction of NANOG expression and an increase of stem-like features in AML cells (Voeltzel et al. 2018).

Additionally, study reported that the secreted stem cell growth factor R-spondin 2 (RSPO2) inhibits BMP signaling to promote self-renewal in AML cells, which acts as a BMP signaling antagonist (Sun et al. 2021). Interestingly, the truncated isoform, SMAD5-beta, was found to have higher expression levels in the undifferentiated CD34^+^ HSCs/LSCs than in the terminally differentiated leukemia, thereby suggesting its implication in stem cell homeostasis. Furthermore, the lack of physical interactions between SMAD5-beta and SMAD4 may represent a novel mechanism to protect pluripotent stem cells and malignant cells from the growth inhibitory and differentiation signals of BMPs (Jiang et al. 2000).

In summary, the role of the BMP signaling in AML is context-dependent, particularly in LSCs. Activation of BMP signaling is necessary for maintaining stemness and promoting AML lineage phenotype production in progenitor cells. Conversely, inhibiting BMP signaling can protect against AML differentiation in specific cellular contexts. These findings underscore the pleiotropic nature of BMP signaling in AML and emphasize the importance of developing precise and personalized therapies for AML in the future.

## BMP signaling in lung cancer

Lung cancer is the leading cause of cancer mortality and accounts for 30% of all deaths from cancer (Jemal et al. 2010; Siegel et al. 2013). Despite advancements in medical care, the prognosis for lung cancer remains poor, with 85% of patients succumbing to the disease. BMP signaling, which is normally absent in adult lung tissue (Sountoulidis et al. 2012), becomes reactivated in lung injury as well as non-small cell lung carcinomas (NSCLC) and small cell carcinomas (Langenfeld et al. 2005). NSCLC exhibits significant overexpression of BMP2 compared to normal lung tissue and benign tumors, and depletion of BMP2 or its receptor BMPR2 has been shown to reduce cell migration and invasiveness (Wu et al. 2022).

Recent studies have shown that the BMP signaling plays a crucial role in promoting lung cancer cell growth and survival (Langenfeld et al. 2013). Downregulation of type I BMP receptors with siRNA or small molecule inhibitors (DMH1, DMH2) in lung cancer cells caused growth inhibition and cell death, while the forced expression of *ID3* attenuated growth suppression and cell death caused by BMP receptor inhibitors. These findings suggest that BMP signaling is a potential therapeutic target for lung cancer treatment (Augeri et al. 2016). Furthermore, combining inhibition of BMP signaling with mitochondrial targeting agents induces AIF (apoptosis-inducing factor) caspase-independent cell death by hyperactivating AMPK, indicating the potential use of this combination as a novel therapeutic strategy for lung cancer treatment (Mondal et al. 2022). Moreover, RUNX2 could recruit histone H3K9-specific methyltransferase Suv39h1 to *BMP3B* (*GDF10*) proximal promoter and then suppress the *BMP3B* expression, which is regarded as a tumor growth inhibitor and a gene silenced in lung cancers (Dai et al. 2004; Tandon et al. 2012).

Taken together, these finding demonstrate that BMP signaling plays an essential role in lung cancer cell growth and survival. BMP signaling inhibitors could present a potential therapeutic target for lung cancer treatment, alone or in combination with other agents. However, further research is needed to investigate the clinical utility of targeting BMP signaling for lung cancer treatment.

## BMP signaling in prostate cancer

Prostate cancer is a significant cause of male cancer-related mortality (Siegel et al. 2016). The interplay between TGF-β and BMP signaling pathways within prostate cancer is intricate, with distinct roles (Lu et al. 2017). Genetic deletions of Tgfbr2 and Bmpr2 in a Pten-null mouse model reveal that TGFβ restrains cancer progression, while BMP signaling drives advancement (Lu et al. 2017). BMP signaling interacts with pathways like WNT and PI3K/AKT, fostering cancer progression and therapy resistance (Chen et al. 2016; Lee et al. 2014a; Murillo-Garzón and Kypta 2017). BMP ligands are key subjects in research on prostate cancer stemness, migration, invasion, growth, and metastasis.

Within the intricate landscape of prostate cancer, the architects of disorder manifest as basal and ductal stem cells, wielding the potential to spark tumorigenesis and invariably contributing to the unsettling specter of tumor recurrence (Choi et al. 2012; Goldstein et al. 2010). Intriguingly, emerging reports cast a spotlight on BMP signaling as a vigilant guardian of stem/progenitor cell preservation nestled within the basal cell enclave. Noteworthy is the fact that taming the tempestuous BMP5 signaling alone showcases the capacity to impede the otherwise relentless march of cancer progression within prostate basal cells, offering a promising ray of hope (Tremblay et al. 2020). Deeper intricacies are unveiled as BMP6 assumes a central role, conducting a sophisticated symphony of migration and invasion within the domain of prostate cancer cells. Amplifying its significance, BMP6 intricately coordinates the heightened expression of MMP and ID1, propelling prostate cancer cells towards an elevated prowess in migration and invasion (Darby et al. 2008).

However, not all BMP ligands assume tumor-promoting roles in prostate cancer; BMP7, in particular, stands as an exception. Initial reports highlighted BMP7’s ability to curtail tumor growth by upregulating CDKN1A in prostate cancers (Miyazaki et al. 2004). BMP7 exercises control over epithelial homeostasis within the human prostate, safeguarding the epithelial phenotype and impeding bone metastases of prostate cancer in vivo (Buijs et al. 2007). Furthermore, BMP7 induces reversible senescence and growth arrest of cancer stem cells (CSCs) both in vitro and in vivo, achieved by upregulating NDRG1 through the p38 pathway in prostate cancer (Kobayashi et al. 2011).

BMP signaling also assumes crucial significance in the context of bone metastases in prostate cancer, a factor responsible for 80% of patient deaths (Ibrahim et al. 2010). In vitro investigations have illuminated the cooperative impact of BMP4 and SHH on fostering the survival of prostate cancer cells alongside the differentiation of bone stromal cells, potentially culminating in the osteoblastic metastasis characteristic of prostate cancer (Nishimori et al. 2012). Moreover, findings from in vivo studies have underscored the involvement of BMP4 in osteogenesis within a xenograft model of prostate cancer bone metastasis. Notably, inhibition of BMP receptors by LDN193189 has been shown to impede osteoblast differentiation and restrain tumor growth (Lee et al. 2011).

In summary, BMP signaling significantly impacts prostate cancer malignancy, with BMP ligands being key factors. Certain BMP ligands maintain cancer stemness, enhance migration and invasion, and drive metastasis. Noteworthy is BMP7’s unique role, reducing prostate tumor growth. These studies illuminate intricate BMP coordination with other pathways, fueling cancer progression and suggesting BMP modulation as a promising therapeutic strategy for curbing prostate tumor advancement.

## BMP signaling in osteosarcoma and chondrosarcoma

BMPs, originally recognized for their bone-forming prowess, play pivotal roles in bone and cartilage development throughout life (Salazar et al. 2016). Notably, disrupted BMP signaling frequently underpins human bone and cartilage disorders, particularly osteosarcomas and chondrosarcoma. These two malignancies, accounting for around 30% of primary bone sarcomas, often exhibit altered BMP presence (Evola et al. 2017). In osteosarcomas, BMPs tend to be linked with less differentiated mesenchymal cells, contributing to an unfavorable prognosis (Nguyen et al. 2014). Malignant dedifferentiated chondrosarcomas also display BMP expression and undifferentiated characteristics. Clinical investigations reveal that osteosarcomas with active BMP signaling exhibit resistance to chemotherapy, heightened metastasis tendencies, and significantly reduced five-year survival rates (Yoshikawa et al. 1988). However, BMP signaling’s role in osteosarcomas is diverse. Patients with BMP-signaling-negative tumors have reported lower overall survival (Mohseny et al. 2012).

Recent studies have delved into the potential impact of BMP signaling on osteosarcoma. An earlier investigation documented the inhibitory prowess of BMP2 in curbing sarcomagenesis within “cancer stem cells” of osteosarcoma. This inquiry pinpointed osteosarcoma stem cells derived from the OS99-1 cell line, displaying elevated ALDH activity, a trait profoundly dampened by BMP2 treatment both in controlled laboratory conditions and in live subjects (Wang et al. 2011). Conversely, an alternate study highlighted the limitations of BMP2/9 overexpression in prompting osteogenic differentiation. In osteosarcomas afflicted with differentiation anomalies, BMP exerted pro-mitogenic effects, revealing a complex interplay between BMP signaling and osteosarcoma progression (Luo et al. 2008). Intriguingly, the exposure of osteosarcoma cells to diverse extracellular matrix (ECM) components, in the presence or absence of BMP2, led to an unexpected revelation. BMP2 emerged as a driver of osteosarcoma cell migration, achieved through its modulation of fibronectin-integrin-β1 signaling pathways (Sotobori et al. 2006).

In summary, BMPs have been extensively studied as osteoinductive molecules, exhibiting documented expression patterns in both benign and malignant bone tumors. However, the effects of BMPs on osteosarcoma and chondrosarcoma biology are diverse and multifaceted. In the context of osteosarcoma, BMP signaling demonstrates a dichotomy of effects. It exerts anti-tumor influences on osteosarcoma cancer stem cells (CSCs), orchestrating transitions from a stemness state to a differentiation state. Simultaneously, BMP signaling can paradoxically stimulate osteosarcoma cell migration and invasion, particularly when certain osteosarcoma cells develop resistance to BMP-induced osteogenic differentiation. This intricate interplay is facilitated through crosstalk with fibronectin-integrin-β1 signaling pathways.

These discoveries provide a foundational framework for evaluating the clinical relevance of BMP signaling in predicting the outcomes of osteosarcoma and chondrosarcoma. Furthermore, they underscore the potential of modulating BMP signaling as a therapeutic avenue for curbing osteosarcomagenesis, inhibiting growth, and thwarting invasive tendencies in these malignancies.

## BMP signaling in cancer metastasis

Tumor metastasis stands as the primary culprit behind cancer-related fatalities. Grasping the intricate molecular mechanisms that underlie this menacing process holds the key to reigning in this formidable ailment. Within the metastatic cascade, numerous signaling pathways choreograph the intricate cellular ballet, encompassing stalwarts such as TGFβ (Massagué 2008), BMP (Ren et al. 2020), PDGF (Nissen et al. 2007), and the JAK/STAT pathways (Yadav et al. 2011).

In the realm of cancer, the TGFβ pathway’s duality has been long acknowledged. Its role wavers between anti-tumor sentinel and pro-metastasis instigator, its inclination hinging upon cellular phenotype, genetic aberrations, and an array of allied factors (Massagué 2008). Similarly, mirroring TGFβ’s enigmatic behavior, BMP engagement with tumor cells showcases a dual face. While initially stifling cellular proliferation, BMP stimulation paradoxically emboldens the machinery of cell migration and invasion, as observed in compelling research (Ketolainen et al. 2010).

BMP signaling frequently intersects with other signaling pathways, sometimes acting as a facilitator of tumor metastasis. Recent investigations have unveiled intriguing insights. Notably, in the context of highly invasive breast cancers, TGFβ signaling has been found to counteract BMP-induced SMAD1/5/8 activation. This interplay leads to a substantial reduction in tumor self-seeding, as well as diminished liver and bone metastasis (Ren et al. 2020). In a related context, the interplay between BMP and SHH pathways forms a cooperative and intricate cycle that fuels the bone metastasis of prostate cancer, as observed in prior studies (Nishimori et al. 2012).In addition, the interwoven connection of BMP and NF-κB signaling pathways emerges as a pivotal driver of both oncogenesis and metastasis in esophageal squamous cell carcinoma, a revelation elucidated through research endeavors (Lau et al. 2017).

Moreover, the activation of BMP signaling within the neighboring tumor microenvironment has been found to potentiate the metastatic dissemination of tumors. Specifically, the stimulation of fibroblasts by BMP can exert diverse effects. In the context of prostate tumors, BMP stimulation of fibroblasts has been demonstrated to foster angiogenesis (Yang et al. 2008). Similarly, when mammary fibroblasts are exposed to BMP stimulation, it leads to an augmentation in tumor cell invasion. This is coupled with an escalation in the secretion of inflammatory cytokines and the remodeling of the extracellular matrix (Owens et al. 2013).

Recent investigations have illuminated the potential of systemic BMP signaling inhibition as a means to halt tumor progression and metastasis, encompassing both the tumor itself and its microenvironment. A noteworthy illustration comes from the use of DMH1, a BMP antagonist, which has exhibited promising outcomes. Treatment with DMH1 has shown the capability to curtail lung metastasis in breast cancer. Additionally, in vivo results displayed a reduction in tumor proliferation and an increase in apoptotic processes, highlighting the potential therapeutic significance of modulating BMP signaling (Owens et al. 2015).

**Fig. 2:**
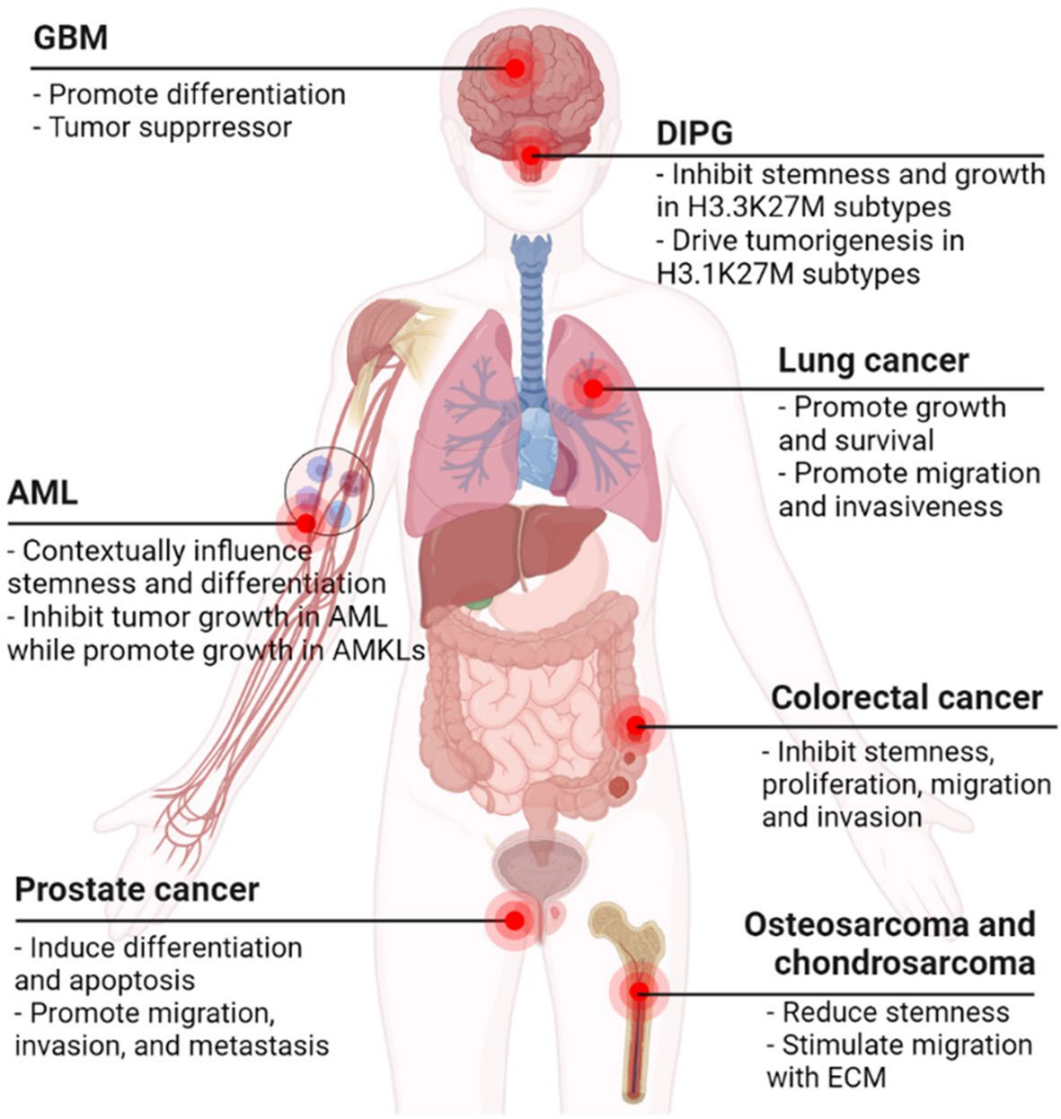
The role of BMP signaling in various human cancers. BMP signaling exhibits context-dependent pleiotropic effects across diverse cancers. In certain cancer types (e.g., lung cancer), BMP signaling can drive tumorigenesis, whereas in others (e.g., GBM), it exerts inhibitory influence on tumor progression. Notably, within distinct tumor subtypes of DIPG and AML, BMP signaling assumes a dual role. Furthermore, the functions of BMP signaling in prostate cancer and osteosarcoma are contingent upon the cellular context, introducing variability in its impact. Created with BioRender.com

Collectively, these investigations substantiate the multifaceted role of BMP signaling in the intricate landscape of cancer evolution and advancement. BMP signaling exhibits a dichotomy, capable of curbing tumor stemness while concurrently fostering the orchestration of organ-specific tumor metastasis (Fig. 2). The intricate interplay between BMP signaling and other prominent pathways serves as a facilitator, steering the course of tumor metastatic spread and overall progression across various cancer types. Emerging as a promising avenue for therapeutic intervention, the restraint of BMP signaling within both the tumor and its encompassing microenvironment emerges as a prospective approach in combatting the specter of future cancer metastasis.

## Conclusions and perspectives

This review provides an overview of the findings from numerous studies that have investigated the function of BMP signaling in cancer stemness and differentiation. Similar to the TGFβ signaling pathway, the role of the BMP pathway in tumorigenesis is complex and varies depending on the specific cellular context, acting as either a tumor suppressor or a tumor promoter.

Understanding the precise mechanisms and the intricate crosstalk between the BMP and TGFβ signaling pathways is of great importance to unravel the complexities of tumorigenesis. While the BMP signaling has been implicated in various aspects of cancer development, including tumor growth, metastasis, and stemness (Table 1 and Fig. 2), there are still many unanswered questions. One such question pertains to the potential overlapping and distinct roles of the BMP and TGFβ pathways in different types of cancers. Further investigation is needed to elucidate the interplay and competitive effects between these two signaling pathways within tumor cells.

Importantly, the activity of BMP signaling is tightly regulated by a plethora of factors, and disrupting this delicate balance can alter the characteristics of normal cells and lead to their transformation into tumor cells (Table 1). Understanding the key factors involved in this regulatory process is crucial for comprehending the development and progression of cancer. In this regard, secreted antagonists play a significant role in the regulatory network of BMP signaling. The tumor microenvironment is enriched with various secreted factors, including BMP signaling antagonists. The concentration and activity of BMP ligands and antagonists may depend on intricate cell-to-cell communication, and it has been suggested that cancer stem cells may secrete BMP signaling antagonists as a means to inhibit the BMP pathway within the tumor microenvironment. Hence, investigating the roles of BMP ligands and antagonists within the tumor microenvironment may provide valuable insights into the regulatory networks that influence cancer development and progression.

BMP ligands introduce further layers of intricacy to the already complex regulatory landscape within different tumors. It’s noteworthy that distinct BMP ligands might execute analogous functions within a given tumor. Paradoxically, a singular BMP ligand could even yield disparate functions when situated in diverse tumor types. As a result, the influence of BMP signaling takes center stage within specific tumor contexts. Delving into the operational mechanisms of these ligands becomes imperative, as it holds the potential to elucidate the exact contribution of the BMP pathway within these specific tumor types.

Given the diverse roles of BMP signaling in cancer, there is considerable potential for the development of novel therapeutic approaches targeting these pathways. In cases where BMP signaling acts as a tumor suppressor, delivering exogenous BMP ligands to tumors using various methods, such as through the use of vaccinia viruses, may hold clinical promise and offer potential benefits to patients. Additionally, targeting BMP signaling pathway antagonists or negative regulators, such as NOG (noggin) and SMAD6, using small molecule inhibitors could effectively promote BMP signaling activity and potentially inhibit tumor growth.

Conversely, in situations where BMP signaling act as tumor promoters, interventions at different levels could be considered. Direct delivery of inhibitors, such as antisense oligonucleotides, specifically targeting BMP ligand production within the tumor, could potentially offer a means to prolong patient survival. Furthermore, inhibiting ligand-receptor interactions using antibodies against BMP ligands or BMP receptors, as well as employing small molecule inhibitors that target BMP receptor kinases, like LDN-193,189, could provide alternative and potentially more effective therapeutic approaches for blocking BMP signaling in tumors.

Lastly, since BMP ligands and antagonists are secreted proteins, the measurement of their concentrations in a patient’s blood or specific tissues may have diagnostic value and could aid in assessing the level of tumorigenesis. Monitoring the levels of these signaling molecules may offer valuable insights into disease progression and guide treatment decisions.

In conclusion, the comprehensive understanding of BMP signaling in cancer is a complex and evolving field. The intricate interplay between BMP, TGFβ and other signaling pathways, the balance of BMP ligands and antagonists in the tumor microenvironment, and the potential for targeted therapeutic interventions make this an area of great interest for future research and the development of personalized cancer therapies.

## Data Availability

Not applicable.

## Abbreviations

BMP: Bone morphogenetic protein
CSC: Cancer stem cell
GBM: Glioblastoma multiforme
GSCs: Glioma stem cells
NSCs: Neural stem cells
FOP: Fibrodysplasia ossificans progressiva
DIPG: Diffuse intrinsic pontine glioma
CRC: Colorectal cancer
AML: Acute myeloid leukemia
LSCs: Leukemia stem cells
HSCs: Hematopoietic stem cells
AMKLs: Acute megakaryoblastic leukemias
NSCLC: Non-small cell lung cell

## References

[CR1] Agius E, Decker Y, Soukkarieh C, Soula C, Cochard P (2010). Role of BMPs in controlling the spatial and temporal origin of GFAP astrocytes in the embryonic spinal cord. Dev Biol.

[CR2] Agnew C, Ayaz P, Kashima R, Loving HS, Ghatpande P, Kung JE (2021). Structural basis for ALK2/BMPR2 receptor complex signaling through kinase domain oligomerization. Nat Commun.

[CR3] Aldape K, Zadeh G, Mansouri S, Reifenberger G, von Deimling A (2015). Glioblastoma: pathology, molecular mechanisms and markers. Acta Neuropathol.

[CR4] Armandari I, Zomerman WW, Plasschaert SLA, Smit MJ, Martini TEI, de Camargo Magalhães ES (2021). CREB signaling activity correlates with differentiation and survival in medulloblastoma. Sci Rep.

[CR5] Atsuta Y, Takahashi Y (2016). Early formation of the mullerian duct is regulated by sequential actions of BMP/Pax2 and FGF/Lim1 signaling. Development.

[CR6] Augeri DJ, Langenfeld E, Castle M, Gilleran JA, Langenfeld J (2016). Inhibition of BMP and of TGFβ receptors downregulates expression of XIAP and TAK1 leading to Lung cancer cell death. Mol Cancer.

[CR7] Bao S, Wu Q, McLendon RE, Hao Y, Shi Q, Hjelmeland AB (2006). Glioma stem cells promote radioresistance by preferential activation of the DNA damage response. Nature.

[CR8] Barker N, van Es JH, Kuipers J, Kujala P, van den Born M, Cozijnsen M (2007). Identification of stem cells in small intestine and colon by marker gene Lgr5. Nature.

[CR9] Batts LE, Polk DB, Dubois RN, Kulessa H (2006). Bmp signaling is required for intestinal growth and morphogenesis. Dev Dyn.

[CR10] Beumer J, Puschhof J, Yengej FY, Zhao L, Martinez-Silgado A, Blotenburg M (2022). BMP gradient along the intestinal villus axis controls zonated enterocyte and goblet cell states. Cell Rep.

[CR11] Bier E, De Robertis EM (2015). BMP gradients: a paradigm for morphogen-mediated developmental patterning. Science.

[CR12] Blanpain C, Fuchs E (2014). Plasticity of epithelial stem cells in tissue regeneration. Science.

[CR13] Bocciardi R, Bordo D, Di Duca M, Di Rocco M, Ravazzolo R (2009). Mutational analysis of the ACVR1 gene in Italian patients affected with fibrodysplasia ossificans progressiva: confirmations and advancements. Eur J Hum Genet.

[CR14] Broderick P, Carvajal-Carmona L, Pittman AM, Webb E, Howarth K, Rowan A (2007). A genome-wide association study shows that common alleles of SMAD7 influence colorectal cancer risk. Nat Genet.

[CR15] Buczkowicz P, Hoeman C, Rakopoulos P, Pajovic S, Letourneau L, Dzamba M (2014). Genomic analysis of diffuse intrinsic pontine gliomas identifies three molecular subgroups and recurrent activating ACVR1 mutations. Nat Genet.

[CR16] Buijs JT, Rentsch CA, van der Horst G, van Civerveld PGM, Wetterwald A, Schwaninger R (2007). BMP7, a putative regulator of epithelial homeostasis in the human prostate, is a potent inhibitor of prostate cancer bone metastasis in vivo. Am J Pathol.

[CR17] Cai J, Pardali E, Sánchez-Duffhues G, ten Dijke P (2012). BMP signaling in vascular diseases. FEBS Lett.

[CR18] Caja L, Tzavlaki K, Dadras MS, Tan EJ, Hatem G, Maturi NP (2018). Snail regulates BMP and TGFβ pathways to control the differentiation status of glioma-initiating cells. Oncogene.

[CR19] Calon A, Espinet E, Palomo-Ponce S, Tauriello DV, Iglesias M, Céspedes MV (2012). Dependency of colorectal cancer on a TGF-β-driven program in stromal cells for metastasis initiation. Cancer Cell.

[CR20] Carvalho D, Taylor KR, Olaciregui NG, Molinari V, Clarke M, Mackay A (2019). ALK2 inhibitors display beneficial effects in preclinical models of ACVR1 mutant diffuse intrinsic pontine glioma. Commun Biol.

[CR21] Chen J, Li Y, Yu TS, McKay RM, Burns DK, Kernie SG (2012). A restricted cell population propagates glioblastoma growth after chemotherapy. Nature.

[CR22] Chen H, Zhou L, Wu X, Li R, Wen J, Sha J (2016). The PI3K/AKT pathway in the pathogenesis of prostate cancer. Front Biosci (Landmark Ed).

[CR23] Chen E, Yang F, He H, Li Q, Zhang W, Xing J (2018). Alteration of tumor suppressor BMP5 in sporadic colorectal cancer: a genomic and transcriptomic profiling based study. Mol Cancer.

[CR24] Choi N, Zhang BY, Zhang L, Ittmann M, Xin L (2012). Adult murine prostate basal and luminal cells are self-sustained lineages that can both serve as targets for prostate cancer initiation. Cancer Cell.

[CR25] Choi YJ, Ingram PN, Yang K, Coffman L, Iyengar M, Bai S (2015). Identifying an ovarian cancer cell hierarchy regulated by bone morphogenetic protein 2. Proc Natl Acad Sci U S A.

[CR26] Clara JA, Monge C, Yang Y, Takebe N (2020). Targeting signalling pathways and the immune microenvironment of cancer stem cells - a clinical update. Nat Rev Clin Oncol.

[CR27] Crispino JD, Le Beau MM (2012). BMP meets AML: induction of BMP signaling by a novel fusion gene promotes pediatric acute leukemia. Cancer Cell.

[CR28] Dai Z, Popkie AP, Zhu WG, Timmers CD, Raval A, Tannehill-Gregg S (2004). Bone morphogenetic protein 3B silencing in non-small-cell lung cancer. Oncogene.

[CR29] Darby S, Cross SS, Brown NJ, Hamdy FC, Robson CN (2008). BMP-6 over-expression in prostate cancer is associated with increased Id-1 protein and a more invasive phenotype. J Pathol.

[CR30] David CJ, Massagué J (2018). Contextual determinants of TGFβ action in development, immunity and cancer. Nat Rev Mol Cell Biol.

[CR31] Davis H, Irshad S, Bansal M, Rafferty H, Boitsova T, Bardella C (2015). Aberrant epithelial GREM1 expression initiates colonic tumorigenesis from cells outside the stem cell niche. Nat Med.

[CR32] Davis H, Raja E, Miyazono K, Tsubakihara Y, Moustakas A (2016). Mechanisms of action of bone morphogenetic proteins in cancer. Cytokine Growth Factor Rev.

[CR33] De Kouchkovsky I, Abdul-Hay M (2016). Acute myeloid leukemia: a comprehensive review and 2016 update. Blood Cancer J.

[CR34] Derynck R, Turley SJ, Akhurst RJ (2021). TGFβ biology in cancer progression and immunotherapy. Nat Rev Clin Oncol.

[CR35] Döhner H, Weisdorf DJ, Bloomfield CD (2015). Acute myeloid leukemia. N Engl J Med.

[CR36] Evola FR, Costarella L, Pavone V, Caff G, Cannavò L, Sessa A (2017). Biomarkers of osteosarcoma, chondrosarcoma, and Ewing Sarcoma. Front Pharmacol.

[CR37] Fontebasso AM, Papillon-Cavanagh S, Schwartzentruber J, Nikbakht H, Gerges N, Fiset PO (2014). Recurrent somatic mutations in ACVR1 in pediatric midline high-grade astrocytoma. Nat Genet.

[CR38] Fortin J, Tian R, Zarrabi I, Hill G, Williams E, Sanchez-Duffhues G (2020). Mutant ACVR1 arrests glial cell differentiation to drive tumorigenesis in pediatric gliomas. Cancer Cell.

[CR39] Fortner RT, Schock H, Jung S, Allen NE, Arslan AA, Brinton LA (2017). Anti-mullerian hormone and endometrial cancer: a multi-cohort study. Br J Cancer.

[CR40] Gaarenstroom T, Hill CS (2014). TGF-beta signaling to chromatin: how smads regulate transcription during self-renewal and differentiation. Semin Cell Dev Biol.

[CR41] Gal H, Amariglio N, Trakhtenbrot L, Jacob-Hirsh J, Margalit O, Avigdor A (2006). Gene expression profiles of AML derived stem cells; similarity to hematopoietic stem cells. Leukemia.

[CR42] Gao H, Chakraborty G, Lee-Lim Ai P, Mo Q, Decker M, Vonica A (2012). The BMP inhibitor Coco reactivates breast cancer cells at lung metastatic sites. Cell.

[CR43] Goldstein AS, Huang JT, Guo CY, Garraway IP, Witte ON (2010). Identification of a cell of origin for human prostate cancer. Science.

[CR44] Gomez-Puerto MC, Iyengar PV, Garcia de Vinuesa A, Ten Dijke P, Sanchez-Duffhues G (2019). Bone morphogenetic protein receptor signal transduction in human disease. J Pathol.

[CR45] Gramann AK, Venkatesan AM, Guerin M, Ceol CJ (2019). Regulation of zebrafish melanocyte development by ligand-dependent BMP signaling. Elife.

[CR46] Gruber TA, Larson Gedman A, Zhang J, Koss CS, Marada S, Ta HQ (2012). An inv(16)(p13.3q24.3)-encoded CBFA2T3-GLIS2 fusion protein defines an aggressive subtype of pediatric acute megakaryoblastic leukemia. Cancer Cell.

[CR47] Guo X, Wang XF (2009). Signaling cross-talk between TGF-beta/BMP and other pathways. Cell Res.

[CR48] Haramis APG (2004). De novo crypt formation and juvenile polyposis on BMP inhibition in mouse intestine. Science.

[CR49] He XC, Zhang JW, Tong WG, Tawfik O, Ross J, Scoville DH (2004). BMP signaling inhibits intestinal stem cell self-renewal through suppression of wnt-beta-catenin signaling. Nat Genet.

[CR50] Hegarty SV, O’Keeffe GW, Sullivan AM (2013). BMP-Smad 1/5/8 signalling in the development of the nervous system. Prog Neurobiol.

[CR51] Hoeman CM, Cordero FJ, Hu G, Misuraca K, Romero MM, Cardona HJ (2019). ACVR1 R206H cooperates with H3.1K27M in promoting diffuse intrinsic pontine glioma pathogenesis. Nat Commun.

[CR52] Houlston RS, Webb E, Broderick P, Pittman AM, Di Bernardo MC, Lubbe S (2008). Meta-analysis of genome-wide association data identifies four new susceptibility loci for colorectal cancer. Nat Genet.

[CR53] Huang P, Chen A, He W, Li Z, Zhang G, Liu Z (2017). BMP-2 induces EMT and Breast cancer stemness through rb and CD44. Cell Death Discov.

[CR54] Husanie H, Abu-Remaileh M, Maroun K, Abu-Tair L, Safadi H, Atlan K (2022). Loss of Tumor suppressor WWOX accelerates Pancreatic cancer development through promotion of TGFβ/BMP2 signaling. Cell Death Dis.

[CR55] Huse M, Chen YG, Massagué J, Kuriyan J (1999). Crystal structure of the cytoplasmic domain of the type I TGF beta receptor in complex with FKBP12. Cell.

[CR56] Ibrahim T, Flamini E, Mercatali L, Sacanna E, Serra P, Amadori D (2010). Pathogenesis of osteoblastic bone metastases from Prostate cancer. Cancer.

[CR57] Ikushima H, Todo T, Ino Y, Takahashi M, Miyazawa K, Miyazono K (2009). Autocrine TGF-beta signaling maintains tumorigenicity of glioma-initiating cells through sry-related HMG-box factors. Cell Stem Cell.

[CR58] Imai Y, Kurokawa M, Izutsu K, Hangaishi A, Maki K, Ogawa S (2001). Mutations of the Smad4 gene in acute myelogeneous Leukemia and their functional implications in leukemogenesis. Oncogene.

[CR59] Jackson CM, Choi J, Lim M (2019). Mechanisms of immunotherapy resistance: lessons from glioblastoma. Nat Immunol.

[CR60] Jaeger E, Leedham S, Lewis A, Segditsas S, Becker M, Cuadrado PR (2012). Hereditary mixed polyposis syndrome is caused by a 40-kb upstream duplication that leads to increased and ectopic expression of the BMP antagonist GREM1. Nat Genet.

[CR61] Jeanpierre S, Nicolini FE, Kaniewski B, Dumontet C, Rimokh R, Puisieux A (2008). BMP4 regulation of human megakaryocytic differentiation is involved in thrombopoietin signaling. Blood.

[CR62] Jemal A, Siegel R, Xu J, Ward E (2010). Cancer statistics, 2010. CA Cancer J Clin.

[CR63] Jia S, Dai F, Wu D, Lin X, Xing C, Xue Y (2012). Protein phosphatase 4 cooperates with smads to promote BMP signaling in dorsoventral patterning of zebrafish embryos. Dev Cell.

[CR64] Jiang Y, Liang H, Guo W, Kottickal LV, Nagarajan L (2000). Differential expression of a novel C-terminally truncated splice form of SMAD5 in hematopoietic stem cells and leukemia. Blood.

[CR65] Jin Y, Park S, Park SY, Lee CY, Eum DY, Shim JW (2022). G9a Knockdown suppresses cancer aggressiveness by facilitating Smad protein phosphorylation through increasing BMP5 expression in luminal a type breast cancer. Int J Mol Sci.

[CR66] Jones C, Karajannis MA, Jones DTW, Kieran MW, Monje M, Baker SJ (2017). Pediatric high-grade glioma: biologically and clinically in need of new thinking. Neuro Oncol.

[CR67] Kaplan FS, Al Mukaddam M, Stanley A, Towler OW, Shore EM (2020). Fibrodysplasia ossificans progressiva (FOP): a disorder of osteochondrogenesis. Bone.

[CR68] Ketolainen JM, Alarmo EL, Tuominen VJ, Kallioniemi A (2010). Parallel inhibition of cell growth and induction of cell migration and invasion in breast cancer cells by bone morphogenetic protein 4. Breast Cancer Res Treat.

[CR69] Khan F, Pang L, Dunterman M, Lesniak MS, Heimberger AB, Chen P (2023). Macrophages and microglia in glioblastoma: heterogeneity, plasticity, and therapy. J Clin Invest.

[CR70] Khuong-Quang D-A, Buczkowicz P, Rakopoulos P, Liu X-Y, Fontebasso AM, Bouffet E (2012). K27M mutation in histone H3.3 defines clinically and biologically distinct subgroups of pediatric diffuse intrinsic pontine gliomas. Acta Neuropathol.

[CR71] Kobayashi A, Okuda H, Xing F, Pandey PR, Watabe M, Hirota S (2011). Bone morphogenetic protein 7 in dormancy and metastasis of prostate cancer stem-like cells in bone. J Exp Med.

[CR72] Kodach LL, Bleurning SA, Musler AR, Peppelenbosch MR, Hommes DW, van Den Brink GR, et al. The bone morphogenetic protein pathway is active in human colon adenomas and inactivated in colorectal cancer. Cancer. 2008a;112(2):300–6. 10.1002/cncr.23160. 10.1002/cncr.23160 18008360

[CR73] Kodach LL, Wiercinska E, De Miranda NFCC, Bleuming SA, Musler AR, Peppelenbosch MP, et al. The bone morphogenetic protein pathway is inactivated in the majority of sporadic colorectal cancers. Gastroenterology. 2008b;134(5):1332–41. 10.1053/j.gastro.2008.02.059. 10.1053/j.gastro.2008.02.059 18471510

[CR74] Kodach LL, Jacobs RJ, Voorneveld PW, Wildenberg ME, Verspaget HW, van Wezel T (2011). Statins augment the chemosensitivity of colorectal cancer cells inducing epigenetic reprogramming and reducing colorectal cancer cell ‘stemness’ via the bone morphogenetic protein pathway. Gut.

[CR75] Kosinski C, Li VSW, Chan ASY, Zhang J, Ho C, Tsui WY (2007). Gene expression patterns of human colon tops and basal crypts and BMP antagonists as intestinal stem cell niche factors. Proc Natl Acad Sci USA.

[CR76] Kraiczy J, McCarthy N, Malagola E, Tie G, Madha S, Boffelli D (2023). Graded BMP signaling within intestinal crypt architecture directs self-organization of the wnt-secreting stem cell niche. Cell Stem Cell.

[CR77] Kreso A, Dick JE (2014). Evolution of the cancer stem cell model. Cell Stem Cell.

[CR78] Krishna S, Choudhury A, Keough MB, Seo K, Ni L, Kakaizada S (2023). Glioblastoma remodelling of human neural circuits decreases survival. Nature.

[CR79] Langenfeld EM, Bojnowski J, Perone J, Langenfeld J (2005). Expression of bone morphogenetic proteins in human lung carcinomas. Ann Thorac Surg.

[CR80] Langenfeld E, Hong CC, Lanke G, Langenfeld J (2013). Bone morphogenetic protein type I receptor antagonists decrease growth and induce cell death of lung cancer cell lines. PLoS ONE.

[CR81] Lau MC, Ng KY, Wong TL, Tong M, Lee TK, Ming XY (2017). FSTL1 promotes metastasis and chemoresistance in esophageal squamous cell carcinoma through NFκB-BMP signaling cross-talk. Cancer Res.

[CR82] Lee J, Son MJ, Woolard K, Donin NM, Li A, Cheng CH (2008). Epigenetic-mediated dysfunction of the bone morphogenetic protein pathway inhibits differentiation of glioblastoma-initiating cells. Cancer Cell.

[CR83] Lee CWL, Ito K, Ito Y (2010). Role of RUNX3 in bone morphogenetic protein signaling in colorectal cancer. Cancer Res.

[CR84] Lee GT, Kwon SJ, Lee JH, Jeon SS, Jang KT, Choi HY (2011). Macrophages induce neuroendocrine differentiation of prostate cancer cells via BMP6-IL6 Loop. Prostate.

[CR85] Lee GT, Kang DI, Ha YS, Jung YS, Chung J, Min K, et al. Prostate cancer bone metastases acquire resistance to androgen deprivation via WNT5A-mediated BMP-6 induction. Br J Cancer. 2014a;110(6):1634–44. 10.1038/bjc.2014.23. 10.1038/bjc.2014.23 PMC3960605 24518599

[CR86] Lee JH, Bhang DH, Beede A, Huang TL, Stripp BR, Bloch KD, et al. Lung stem cell differentiation in mice directed by endothelial cells via a BMP4-NFATc1-thrombospondin-1 axis. Cell. 2014b;156(3):440–55. 10.1016/j.cell.2013.12.039. 10.1016/j.cell.2013.12.039 PMC3951122 24485453

[CR87] Li Y, Liu Y, Liu B, Wang J, Wei S, Qi Z (2018). A growth factor-free culture system underscores the coordination between wnt and BMP signaling in Lgr5(+) intestinal stem cell maintenance. Cell Discov.

[CR88] Liu A, Niswander LA (2005). Bone morphogenetic protein signalling and vertebrate nervous system development. Nat Rev Neurosci.

[CR89] Liu S, Ren J, Ten Dijke P (2021). Targeting TGFβ signal transduction for cancer therapy. Signal Transduct Target Ther.

[CR90] Lombardo Y, Scopelliti A, Cammareri P, Todaro M, Iovino F, Ricci-Vitiani L (2011). Bone morphogenetic protein 4 induces differentiation of colorectal cancer stem cells and increases their response to chemotherapy in mice. Gastroenterology.

[CR91] Lu X, Jin EJ, Cheng X, Feng S, Shang X, Deng P (2017). Opposing roles of TGFbeta and BMP signaling in prostate cancer development. Genes Dev.

[CR92] Luo X, Chen J, Song WX, Tang N, Luo J, Deng ZL (2008). Osteogenic BMPs promote tumor growth of human osteosarcomas that harbor differentiation defects. Lab Invest.

[CR93] Mahmoudi R, Afshar S, Amini R, Jalali A, Saidijam M, Najafi R (2023). Evaluation of BMP-2 as a differentiating and radiosensitizing agent for colorectal cancer stem cells. Curr Stem Cell Res Ther.

[CR94] Martínez VG, Rubio C, Martínez-Fernández M, Segovia C, López-Calderón F, Garín MI (2017). BMP4 induces M2 macrophage polarization and favors tumor progression in bladder cancer. Clin Cancer Res.

[CR95] Massagué J (2008). TGFbeta in cancer. Cell.

[CR96] Massagué J (2012). TGFbeta signalling in context. Nat Rev Mol Cell Biol.

[CR97] Massagué J, Seoane J, Wotton D (2005). Smad transcription factors. Genes Dev.

[CR98] McCarthy N, Manieri E, Storm EE, Saadatpour A, Luoma AM, Kapoor VN (2020). Distinct mesenchymal cell populations generate the essential intestinal BMP signaling gradient. Cell Stem Cell.

[CR99] Miska J, Chandel NS (2023). Targeting fatty acid metabolism in glioblastoma. J Clin Invest.

[CR100] Miyazaki H, Watabe T, Kitamura T, Miyazono K (2004). BMP signals inhibit proliferation and in vivo tumor growth of androgen-insensitive prostate carcinoma cells. Oncogene.

[CR101] Mohseny AB, Cai Y, Kuijjer M, Xiao W, van den Akker B, de Andrea CE (2012). The activities of Smad and gli mediated signalling pathways in high-grade conventional osteosarcoma. Eur J Cancer.

[CR102] Mondal A, Roberge J, Gilleran J, Peng Y, Jia D, Akel M (2022). Bone morphogenetic protein inhibitors and mitochondria targeting agents synergistically induce apoptosis-inducing factor (AIF) caspase-independent cell death in Lung cancer cells. Cell Commun Signal.

[CR103] Morrell NW, Bloch DB, ten Dijke P, Goumans MJ, Hata A, Smith J (2016). Targeting BMP signalling in Cardiovascular Disease and anaemia. Nat Rev Cardiol.

[CR104] Murillo-Garzón V, Kypta R (2017). WNT signalling in prostate cancer. Nat Rev Urol.

[CR105] Nagaraja S, Quezada MA, Gillespie SM, Arzt M, Lennon JJ, Woo PJ (2019). Histone variant and cell context determine H3K27M reprogramming of the enhancer landscape and oncogenic state. Mol Cell.

[CR106] Nguyen A, Scott MA, Dry SM, James AW (2014). Roles of bone morphogenetic protein signaling in osteosarcoma. Int Orthop.

[CR107] Nishimori H, Ehata S, Suzuki HI, Katsuno Y, Miyazono K (2012). Prostate cancer cells and bone stromal cells mutually interact with each other through bone morphogenetic protein-mediated signals. J Biol Chem.

[CR108] Nissen LJ, Cao R, Hedlund EM, Wang Z, Zhao X, Wetterskog D (2007). Angiogenic factors FGF2 and PDGF-BB synergistically promote murine tumor neovascularization and metastasis. J Clin Invest.

[CR109] Owens P, Polikowsky H, Pickup MW, Gorska AE, Jovanovic B, Shaw AK (2013). Bone morphogenetic proteins stimulate mammary fibroblasts to promote mammary carcinoma cell invasion. PLoS ONE.

[CR110] Owens P, Pickup MW, Novitskiy SV, Giltnane JM, Gorska AE, Hopkins CR (2015). Inhibition of BMP signaling suppresses metastasis in mammary cancer. Oncogene.

[CR111] Palencia-Desai S, Rost MS, Schumacher JA, Ton QV, Craig MP, Baltrunaite K (2015). Myocardium and BMP signaling are required for endocardial differentiation. Development.

[CR112] Piccirillo SG, Reynolds BA, Zanetti N, Lamorte G, Binda E, Broggi G (2006). Bone morphogenetic proteins inhibit the tumorigenic potential of human brain tumour-initiating cells. Nature.

[CR113] Porcù E, Maule F, Boso D, Rampazzo E, Barbieri V, Zuccolotto G (2018). BMP9 counteracts the tumorigenic and pro-angiogenic potential of glioblastoma. Cell Death Differ.

[CR114] Qi Z, Li YH, Zhao B, Xu C, Liu Y, Li HN (2017). BMP restricts stemness of intestinal Lgr5(+) stem cells by directly suppressing their signature genes. Nature Commun.

[CR115] Raja E, Komuro A, Tanabe R, Sakai S, Ino Y, Saito N (2017). Bone morphogenetic protein signaling mediated by ALK-2 and DLX2 regulates apoptosis in glioma-initiating cells. Oncogene.

[CR116] Ranjan T, Sengupta S, Glantz MJ, Green RM, Yu A, Aregawi D (2023). Cancer stem cell assay-guided chemotherapy improves survival of patients with recurrent glioblastoma in a randomized trial. Cell Rep Med.

[CR117] Raymond A, Liu B, Liang H, Wei C, Guindani M, Lu Y (2014). A role for BMP-induced homeobox gene MIXL1 in acute myelogenous Leukemia and identification of type I BMP receptor as a potential target for therapy. Oncotarget.

[CR118] Ren J, Wang Y, Ware T, Iaria J, Ten Dijke P, Zhu HJ (2020). Reactivation of BMP signaling by suboptimal concentrations of MEK inhibitor and FK506 reduces organ-specific breast cancer metastasis. Cancer Lett.

[CR119] Salazar VS, Gamer LW, Rosen V (2016). BMP signalling in skeletal development, disease and repair. Nat Rev Endocrinol.

[CR120] Savary K, Caglayan D, Caja L, Tzavlaki K, Bin Nayeem S, Bergstrom T (2013). Snail depletes the tumorigenic potential of glioblastoma. Oncogene.

[CR121] Shen Q, Little SC, Xu M, Haupt J, Ast C, Katagiri T (2009). The fibrodysplasia ossificans progressiva R206H ACVR1 mutation activates BMP-independent chondrogenesis and zebrafish embryo ventralization. J Clin Investig.

[CR122] Shen T, Sun C, Zhang Z, Xu N, Duan X, Feng XH (2014). Specific control of BMP signaling and mesenchymal differentiation by cytoplasmic phosphatase PPM1H. Cell Res.

[CR123] Sherry MM, Reeves A, Wu JK, Cochran BH (2009). STAT3 is required for proliferation and maintenance of multipotency in glioblastoma stem cells. Stem Cells.

[CR124] Shibue T, Weinberg RA (2017). EMT, CSCs, and drug resistance: the mechanistic link and clinical implications. Nat Rev Clin Oncol.

[CR125] Shore EM, Xu M, Feldman GJ, Fenstermacher DA, Cho T-J, Choi IH (2006). A recurrent mutation in the BMP type I receptor ACVR1 causes inherited and sporadic fibrodysplasia ossificans progressiva. Nat Genet.

[CR126] Siegel RL, Naishadham D, Jemal A (2013). Cancer statistics. CA Cancer J Clin.

[CR127] Siegel RL, Miller KD, Jemal A (2016). Cancer statistics 2016. CA Cancer J Clin.

[CR128] Singh SK, Hawkins C, Clarke ID, Squire JA, Bayani J, Hide T (2004). Identification of human brain tumour initiating cells. Nature.

[CR129] Sotobori T, Ueda T, Myoui A, Yoshioka K, Nakasaki M, Yoshikawa H (2006). Bone morphogenetic protein-2 promotes the haptotactic migration of murine osteoblastic and osteosarcoma cells by enhancing incorporation of integrin beta1 into lipid rafts. Exp Cell Res.

[CR130] Sountoulidis A, Stavropoulos A, Giaglis S, Apostolou E, Monteiro R, Chuva de Sousa Lopes SM (2012). Activation of the canonical bone morphogenetic protein (BMP) pathway during lung morphogenesis and adult lung tissue repair. PLoS ONE.

[CR131] Stevens ML, Chaturvedi P, Rankin SA, Macdonald M, Jagannathan S, Yukawa M (2017). Genomic integration of Wnt/beta-catenin and BMP/Smad1 signaling coordinates foregut and hindgut transcriptional programs. Development.

[CR132] Stzepourginski I, Nigro G, Jacob JM, Dulauroy S, Sansonetti PJ, Eberl G (2017). CD34 + mesenchymal cells are a major component of the intestinal stem cells niche at homeostasis and after injury. Proc Natl Acad Sci U S A.

[CR133] Sun R, He L, Lee H, Glinka A, Andresen C, Hübschmann D (2021). RSPO2 inhibits BMP signaling to promote self-renewal in acute Myeloid Leukemia. Cell Rep.

[CR134] Sun Y, Yan K, Wang Y, Xu C, Wang D, Zhou W (2022). Context-dependent tumor-suppressive BMP signaling in diffuse intrinsic pontine glioma regulates stemness through epigenetic regulation of CXXC5. Nat Cancer.

[CR135] Takebe N, Miele L, Harris PJ, Jeong W, Bando H, Kahn M (2015). Targeting notch, hedgehog, and wnt pathways in cancer stem cells: clinical update. Nat Rev Clin Oncol.

[CR136] Tandon M, Gokul K, Ali SA, Chen Z, Lian J, Stein GS (2012). Runx2 mediates epigenetic silencing of the bone morphogenetic protein-3B (BMP-3B/GDF10) in Lung cancer cells. Mol Cancer.

[CR137] Tate CM, Pallini R, Ricci-Vitiani L, Dowless M, Shiyanova T, D’Alessandris GQ (2012). A BMP7 variant inhibits the tumorigenic potential of glioblastoma stem-like cells. Cell Death Differ.

[CR138] Taylor KR, Mackay A, Truffaux N, Butterfield Y, Morozova O, Philippe C, et al. Recurrent activating ACVR1 mutations in diffuse intrinsic pontine glioma. Nat Genet. 2014a;46(5):457–61. 10.1038/ng.2925. 10.1038/ng.2925 PMC4018681 24705252

[CR139] Taylor KR, Vinci M, Bullock AN, Jones C. ACVR1 mutations in DIPG: lessons learned from FOP. Cancer Res. 2014b;74(17):4565–70. 10.1158/0008-5472.Can-14-1298. 10.1158/0008-5472.CAN-14-1298 PMC4154859 25136070

[CR140] Tremblay M, Viala S, Shafer MER, Graham-Paquin AL, Liu C, Bouchard M (2020). Regulation of stem/progenitor cell maintenance by BMP5 in prostate homeostasis and cancer initiation. Elife.

[CR141] Tso JL, Yang S, Menjivar JC, Yamada K, Zhang Y, Hong I (2015). Bone morphogenetic protein 7 sensitizes O6-methylguanine methyltransferase expressing-glioblastoma stem cells to clinically relevant dose of temozolomide. Mol Cancer.

[CR142] van den Brink GR, Offerhaus GJ (2007). The morphogenetic code and colon cancer development. Cancer Cell.

[CR143] Venkatesan AM, Vyas R, Gramann AK, Dresser K, Gujja S, Bhatnagar S (2018). Ligand-activated BMP signaling inhibits cell differentiation and death to promote melanoma. J Clin Invest.

[CR144] Veschi V, Mangiapane LR, Nicotra A, Di Franco S, Scavo E, Apuzzo T (2020). Targeting chemoresistant colorectal cancer via systemic administration of a BMP7 variant. Oncogene.

[CR145] Vescovi AL, Galli R, Reynolds BA (2006). Brain tumour stem cells. Nat Rev Cancer.

[CR146] Voeltzel T, Flores-Violante M, Zylbersztejn F, Lefort S, Billandon M, Jeanpierre S (2018). A new signaling cascade linking BMP4, BMPR1A, ∆Np73 and NANOG impacts on stem-like human cell properties and patient outcome. Cell Death Dis.

[CR147] Walsh DW, Godson C, Brazil DP, Martin F (2010). Extracellular BMP-antagonist regulation in development and Disease: tied up in knots. Trends Cell Biol.

[CR148] Walton KD, Whidden M, Kolterud A, Shoffner SK, Czerwinski MJ, Kushwaha J (2016). Villification in the mouse: Bmp signals control intestinal villus patterning. Development.

[CR149] Wang S, Chen YG (2018). BMP signaling in homeostasis, transformation and inflammatory response of intestinal epithelium. Sci China Life Sci.

[CR150] Wang N, Kim HG, Cotta CV, Wan M, Tang Y, Klug CA (2006). TGFbeta/BMP inhibits the bone marrow transformation capability of Hoxa9 by repressing its DNA-binding ability. EMBO J.

[CR151] Wang L, Park P, Zhang H, La Marca F, Claeson A, Valdivia J (2011). BMP-2 inhibits the tumorigenicity of cancer stem cells in human osteosarcoma OS99-1 cell line. Cancer Biol Ther.

[CR152] Wang Y, Zhu P, Luo J, Wang J, Liu Z, Wu W (2019). LncRNA HAND2-AS1 promotes Liver cancer stem cell self-renewal via BMP signaling. EMBO J.

[CR153] Wisnieski F, Leal MF, Calcagno DQ, Santos LC, Gigek CO, Chen ES (2017). BMP8B is a Tumor suppressor gene regulated by histone acetylation in gastric Cancer. J Cell Biochem.

[CR154] Wong ET, Hess KR, Gleason MJ, Jaeckle KA, Kyritsis AP, Prados MD (1999). Outcomes and prognostic factors in recurrent glioma patients enrolled onto phase II clinical trials. J Clin Oncol.

[CR155] Wu G, Broniscer A, McEachron TA, Lu C, Paugh BS, Becksfort J (2012). Somatic histone H3 alterations in pediatric diffuse intrinsic pontine gliomas and non-brainstem glioblastomas. Nat Genet.

[CR156] Wu G, Diaz AK, Paugh BS, Rankin SL, Ju B, Li Y (2014). The genomic landscape of diffuse intrinsic pontine glioma and pediatric non-brainstem high-grade glioma. Nat Genet.

[CR157] Wu M, Chen G, Li YP (2016). TGF-beta and BMP signaling in osteoblast, skeletal development, and bone formation, homeostasis and Disease. Bone Res.

[CR158] Wu CK, Wei MT, Wu HC, Wu CL, Wu CJ, Liaw H (2022). BMP2 promotes lung adenocarcinoma metastasis through BMP receptor 2-mediated SMAD1/5 activation. Sci Rep.

[CR159] Yadav A, Kumar B, Datta J, Teknos TN, Kumar P (2011). IL-6 promotes head and neck Tumor Metastasis by inducing epithelial-mesenchymal transition via the JAK-STAT3-SNAIL signaling pathway. Mol Cancer Res.

[CR160] Yan H, Zhu S, Song C, Liu N, Kang J (2012). Bone morphogenetic protein (BMP) signaling regulates mitotic checkpoint protein levels in human Breast cancer cells. Cell Signal.

[CR161] Yan K, Wu Q, Yan DH, Lee CH, Rahim N, Tritschler I (2014). Glioma cancer stem cells secrete Gremlin1 to promote their maintenance within the tumor hierarchy. Genes Dev.

[CR162] Yang S, Pham LK, Liao CP, Frenkel B, Reddi AH, Roy-Burman P (2008). A novel bone morphogenetic protein signaling in heterotypic cell interactions in Prostate cancer. Cancer Res.

[CR163] Yang L, Shi P, Zhao G, Xu J, Peng W, Zhang J (2020). Targeting cancer stem cell pathways for cancer therapy. Signal Transduct Target Ther.

[CR164] Yokoyama Y, Watanabe T, Tamura Y, Hashizume Y, Miyazono K, Ehata S (2017). Autocrine BMP-4 signaling is a therapeutic target in colorectal cancer. Cancer Res.

[CR165] Yoshikawa H, Takaoka K, Masuhara K, Ono K, Sakamoto Y (1988). Prognostic significance of bone morphogenetic activity in osteosarcoma tissue. Cancer.

[CR166] Zanconato F, Cordenonsi M, Piccolo S (2016). YAP/TAZ at the roots of cancer. Cancer Cell.

[CR167] Zhang Y, Que J (2020). BMP signaling in development, stem cells, and diseases of the gastrointestinal tract. Annu Rev Physiol.

[CR168] Zhang G, Huang P, Chen A, He W, Li Z, Liu G (2018). How BMP-2 induces EMT and breast cancer stemness through rb and CD44?. Cell Death Dis.

[CR169] Zhou J, Wulfkuhle J, Zhang H, Gu P, Yang Y, Deng J (2007). Activation of the PTEN/mTOR/STAT3 pathway in Breast cancer stem-like cells is required for viability and maintenance. Proc Natl Acad Sci U S A.

[CR170] Zinski J, Tajer B, Mullins MC. TGF-β Family Signaling in Early Vertebrate Development. Cold Spring Harb Perspect Biol. 2018;10(6). 10.1101/cshperspect.a033274. 10.1101/cshperspect.a033274 PMC5983195 28600394

